# Crossover from Ferroelectric to Relaxor Behavior in Ba_1−*x*_Ca*_x_*TiO_3_ (*x* = 0.17) System

**DOI:** 10.3390/ma13122854

**Published:** 2020-06-25

**Authors:** Edita Palaimiene, Jan Macutkevic, Juras Banys, Antoni Winiarski, Irena Gruszka, Janusz Koperski, Andrzej Molak

**Affiliations:** 1 Institute of Applied Electrodynamics and Telecommunications, Vilnius University, Sauletekio av. 3, LT-10257 Vilnius, Lithuania; edita.palaimiene@ff.vu.lt (E.P.); juras.banys@ff.vu.lt (J.B.); 2 Institute of Physics, University of Silesia, ul. 75 Pułku Piechoty 1, PL-40-500 Chorzów, Poland; antoni.winiarski@us.edu.pl (A.W.); irena.gruszka@us.edu.pl (I.G.); janusz.koperski@us.edu.pl (J.K.); andrzej.molak@us.edu.pl (A.M.)

**Keywords:** BaTiO_3_, relaxors, ferroelectrics, dielectric permittivity

## Abstract

The dielectric properties of Ba_1−*x*_Ca*_x_*TiO_3_ (*x* = 0.17) ceramics were studied in a wide frequency range of 20 Hz–53 GHz. Diffused ferroelectric phase transition was revealed close to 339 K in the dielectric properties of ceramics. The behaviour of distributions of relaxation times in vicinity of the ferroelectric phase transition temperature is also typical for order-disorder ferroelectric phase transition. However, at lower temperatures (below 200 K), the most probable relaxation increased according to the Arrhenius law. At lower temperatures the maximum of the imaginary part of dielectric permittivity versus temperature strongly shifted to higher temperatures when the frequency increased (from 125 K at 1.21 kHz to 300 K at 33 GHz). This behaviour was attributed to the dynamics of Ti ions. The origin of the crossover from ferroelectric to relaxor behaviour of Ba_1−*x*_Ca*_x_*TiO_3_ (*x* = 0.17) ceramics is discussed in the paper.

## 1. Introduction

BaTiO_3_ is a ferroelectric and piezoelectric material; its dielectric permittivity at room temperature is about several thousand [1]. Three phase transitions are observed in BaTiO_3_: the first one from cubic paraelectric (PE) to tetragonal ferroelectric (FE) at 398 K, the second one below room temperature (at 273 K) from tetragonal to orthorhombic and the third phase transition from orthorhombic to rhombohedral (at 183 K) [2]. The phase transition types in BaTiO_3_ rather are mixed type, order-disorder and displacive, and the corresponding dielectric dispersion occurs mainly in the terahertz and infrared frequency range [3]. However, for practical applications, the main drawback of BaTiO_3_ is the marked temperature dependence of their dielectric and piezoelectric properties at room temperature. Therefore, various mixed systems based on BaTiO_3_ are investigated very often [4]. Moreover, it is expected that various mixed BaTiO_3_ based systems can substitute lead based piezoelectrics in various applications [5,6,7]. A variety of FE phase transition smearing scenarios are possible by the substitution of several ions in the BaTiO_3_ lattice [8,9,10]. At a high substitution level, relaxor-like behaviour is usually observed in these systems [8,9,10]; however, dipolar glass behaviour was discovered in BaZr_0.5_Ti_0.5_O_3_ [11].

CaTiO_3_ is an incipient FE material with an orthorhombic structure [12]. It was determined that in the Ba_1−*x*_Ca*_x_*TiO_3_ (BCT) system, the highest value of the Curie temperature (*T*_C_), the dielectric permittivity at *T*_C_ and the best piezoelectric properties are observed close to *x* = 0.2 [13]. The piezoelectric constant of the system reaches 620 pC/N, which is higher than reported for Pb(ZrTi)O_3_ [14]. Moreover, the structure of Ba_1−*x*_Ca*_x_*TiO_3_ at room temperature is tetragonal for *x* ≤ 0.2, tetragonal + orthorhombic for 0.4 ≤ *x* ≤ 0.8 and orthorhombic for 0.9 ≤ *x* [13]. Nevertheless, the phase diagram of Ba_1−*x*_Ca*_x_*TiO_3_ was not studied in detail, particularly the dielectric investigations of this system were performed only at low frequencies (below 2 MHz) [13,15,16,17]. The electrical features of BCT were extensively studied for the BCT compounds, which showed transition (DPT) between the FE and PE phases in the vicinity of 400 K. This transition was diffused, showing deviation from classical Curie-Weiss behaviour of dielectric permittivity [17]. Moreover, the low temperature FE–FE transition, which shows the step-like diffused dielectric permittivity anomaly, shifts from 300 to 100 K when the Ca content increases up to *x* = 0.20. The features markedly affected by the Ca ions doping are still not understood [17,18,19,20].

The origins of these phase transitions’ diffusivity, deduced from dielectric permittivity studies, are controversial. The influence of local inhomogeneities, especially in the Ca/Ba sublattice was a matter of discussion. The stability of the FE phase was related to Ca ions doping. The FE–PE transition temperature was weakly affected by the doping, while the FE–FE transition markedly shifted towards a lower temperature [16,17,21,22,23,24]. The occurrence of DPT was attributed to local disorder related to the Ba/Ca sublattice and oxygen vacancies. Residual strain-stress effects were considered because of a large difference between ionic radii of Ba and Ca ions. Hence, the stress could manifest at interfaces between Ba- and Ca-rich areas [25]. Moreover, such micro-heterogeneity would be responsible for the dielectric relaxation, related either to relaxor or to dipolar glass features. The dielectric relaxation, which occurs in the microwave range, would be attributed to Ti ion dynamics; that is, Ti ions hopping between potential wells within the oxygen octahedron [21,26]. This effect might correspond to site occupancy of Ca^2+^ ions, despite the fact that isovalent Ca_Ba_ substitution is preferred. However, minor substitution to the Ti^4+^ ion sublattice, Ca_Ti_, also is possible, requiring charge compensation via oxygen vacancies, *V*_O_^●●^. Therefore, the dynamics of Ti ions could be influenced in such a case [20,27,28]. 

The occurrence of DPT in BCT can depend on the structural inhomogeneity, the grain size and the local non-stoichiometry that relates to technology procedures. The solid state reaction conducted via high temperature sintering can induce substitution disordering. The standard procedure to obtain BCT was one calcination followed by final sintering. It is worth mentioning that better homogenisation of the ion distribution in the Ba/Ca sublattice was achieved by repeated calcination cycles. However, DPT features that indicated another background of the diffusivity were not removed [24]. The wet chemistry provided nano-sized grains of different morphology and crystal lattice and/or surface strain. However, the subsequent high temperature sintering was performed to obtain ceramics pellets for dielectric tests [15,23]. It should be mentioned that intentionally non-stoichiometric BCT ceramics, measured by the cationic ratio α = (Ba + Ca)/Ti, exhibited markedly affected FE–PE phase transition. The phase transition was shifted downward by several dozens of K and markedly diffused [16].

In this work, we tried tuning the dielectric properties convenient for application in the 300–400 K range. Therefore, we modified the technology of preparation of BCT ceramics. BCT was sintered in three steps, at different temperatures, instead of the conventional one calcination followed by one sintering. We would like to mention, in accordance with the literature, that repeated high temperature sintering would lead to increased diffusion of the ions in lattice [24], compensated by oxygen vacancies, are to be presumed. We obtained BCT ceramics, which showed a diffused peak related to FE–PE phase transition and a step like anomaly in permittivity in the lower temperature range. Broadband dielectric investigations were needed in order to discriminate various phases of disordered dielectrics, like dipolar glasses, ferroelectric relaxor, and others [29]. Therefore, the aim of this paper was to study the phase transition features of Ba_0.83_Ca_0.17_TiO_3_ via broadband dielectric spectroscopy.

## 2. Materials and Methods

### 2.1. Ceramics Preparation 

BCT ceramics were sintered using the standard high temperature solid state reaction procedure. The material was sintered from TiO_2_ oxide and carbonates, BaCO_3_ and CaCO_3_, (Sigma-Aldrich Chemie GmbH, Steinheim, Germany, purity ≥ 99%) at ambient air conditions, and modified with respect to the literature reports [16,17]. A Mixing Mill (MM200, Retsch GmbH, Haan, Germany) was applied for blending powders of BaCO_3_, CaCO_3_ and TiO_2_. The raw material was dry milled at 11.5 Hz for 1.5 h. It was pressed at 28 MPa to form pellets and then calcined at 1223 K for 4 h. It was crushed, milled at 13.5 Hz for 1.5 h, pressed, and sintered at 1523 K for 4 h. Then, the pellets were crushed again, milled at 14 Hz for 1.5 h, pressed, and finally sintered at 1673 K for 3 h. The ceramics pellets were cut and samples were polished and ultrasonically washed in distilled water.

### 2.2. XRD (X-Ray Diffraction) Research

The powdered samples were studied on an Empyrean X-ray powder diffractometer (type 9430 060 03001, PANalytical, Malvern, UK) using filtered CuK_α_ radiation (*λ* = 0.154056 nm; *U* = 40 kV, *I* = 30 mA) and the *θ*-*θ* scan technique. The diffraction pattern was collected at room temperature, in the 2*θ* range (10°–100°) with a step size of 0.0130° (2*θ*). The scan step time was 1450 s. A PIXcel^3D^ detector (Malvern Panalytical, Malvern, UK) was used. The patterns were obtained for powdered ceramics. Phase identification and crystal structure investigations were performed using the HighScore Plus software and powder diffraction data base PDF-4+. The HighScore Plus program of PANalytical (currently Malvern Panalytical), the Rietveld method for refining the structure, and the pseudo-Voigt function to analyze the line profile were applied [30].

### 2.3. Microanalysis

A scanning microscope JSM-5410 (JEOL, Tokyo, Japan) equipped with an energy dispersion X-ray spectrometer, itself equipped with a Si(Li) X-ray detector, was applied to determine the morphology and chemical composition. The vacuum in the test chamber was 10^−4^–10^−5^ Pa. The chemical composition was estimated using ISIS-300SEMQuant software (JEOL). The measuring error was of the order of 1%. Secondary electron images (SEI) and backscattered electrons images (BEI) were collected from fractured BCT ceramics covered with Au. Chemical composition was determined from several areas. We conducted measurements to estimate chemical composition and its fluctuation, depending on the state of the ceramics’ surface (cleaved polished surface, cleaved un-polished surface), large area (magnification ×1000), small area (magnification ×7500) and point measurement on individual grain (beam diameter ~1 µm).

### 2.4. BDS (Broadband Dielectric Spectroscopy)

Broadband dielectric spectroscopy (BDS) measurements were conducted using several techniques. At low frequencies (20 Hz–1 MHz), the real and the imaginary part of the complex dielectric permittivity was obtained from the complex capacitance, which was measured by a precision HP4824 LCR meter (Hewlett Packard, Palo Alto, CA, USA). At frequencies from 1 MHz to 1 GHz, the complex dielectric permittivity was calculated from the complex reflection coefficient, which was measured by an Agilent 8714ET vector network analyser (Agilient Technologies, Santa Clara, CA, USA), using a sample placed in a coaxial line. In the microwave frequency range, from 8 GHz to 12 GHz, the reflectance and the transmission of a thin dielectric rod placed inside a waveguide were studied. For these measurements, a custom-made waveguide spectrometer was used [31]. The typical value for the rod diameter was several hundred micrometers. In the frequency range from 1 MHz to 12 GHz, the measurement accuracy was ~10%. All measurements also were conducted in the temperature range of 100–500 K. Silver paste was used to make electric contact with the samples.

## 3. Results

### 3.1. XRD

**Figure 1:**
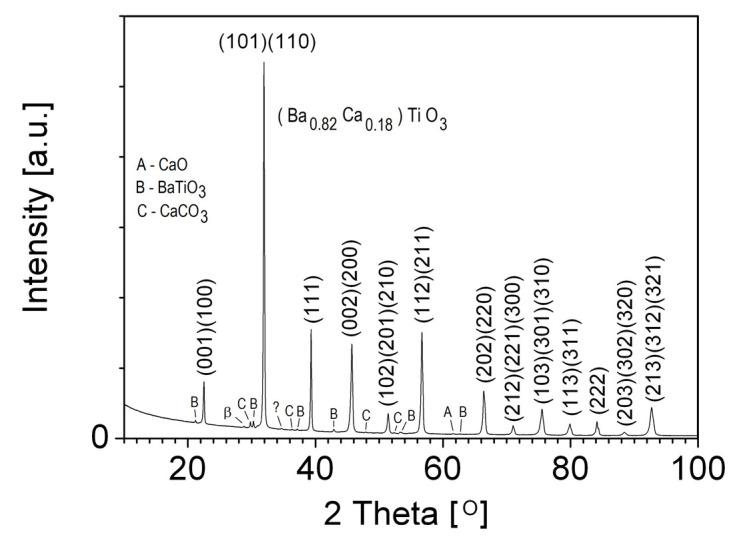
XRD (X-ray diffraction) pattern obtained for Ba_0.82_Ca_0.18_TiO_3_ ceramics.

The XRD pattern of BCT was used for the identification of phases (Figure 1). We found a predominant BCT tetragonal phase with the space group *P*4*mm.* Ba_0.88_Ca_0.12_TiO_3_ compound (No. 01-081-0042 in the base PDF-4+) has the same space group as obtained in the BCT phase, therefore it was taken as the starting compound for the fitting procedure. The Rietveld fitting was performed for *x* = 0.17, 0.18, and 0.20. The best fit (*R*_Bragg_ = 6.06) of the measured and the calculated patterns was obtained for *x* = 0.18. The elementary cell volume decreased when the Ca ion content increased due to a Ca ionic radius (*R* = 1.34 Å) that is smaller than the Ba ionic radius (*R* = 1.61 Å). Crystal lattice parameters are shown in Table 1 for comparison. The crystal lattice structure of BCT was satisfactorily determined at room temperature and the tetragonal phase was consistent with FE properties. 

In addition, small amounts of three other phases: CaO, BaTiO_3_, and CaCO_3_ were found. The concentration of these three phases was less than 2%. Moreover, sharp diffraction lines from these compounds indicated that these compounds probably separated at grain boundaries. The lines in the pattern, related to the CaO, BaTiO_3_, and CaCO_3_ phases, were identified and labelled with A, B, and C (see Figure 1). The cubic $Fm\bar{3}m$, tetragonal $Pm\bar{3}m$, and monoclinic *P2_1_/c* space groups were determined, respectively. The line which was not identified is labeled as “question mark, ?”. The *K*_β_ line of BCT *hkl* reflex (101)(110), also was discerned in the XRD pattern (label: β). 

The absence of a superstructure line in the X-ray diffraction pattern, in low angle range, indicated a random exchange of barium atoms by calcium atoms. The Ruddlesden-Popper phase, which might be related to disturbed stoichiometry and which consists of multiple alkali metal ion oxides in the surface layer, was not detected [32,33]. The small amount of precipitation of the oxides, on pico-scale level, would not be detected using the performed XRD test.

### 3.2. SEM (Scanning Electron Microscope)

**Figure 2:**
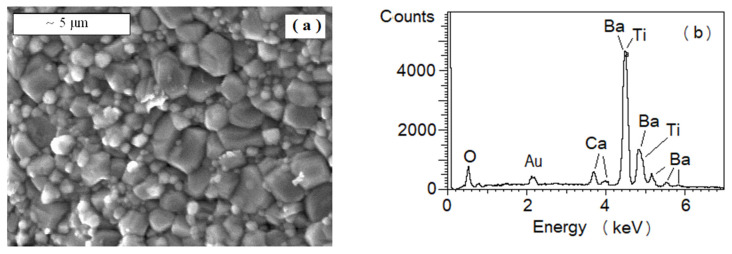
(**a**) SEM map obtained for BCT ceramics (magnification: 10,000). (**b**) Survey spectrum of BCT ceramics.

The SEM images of the BCT fractured ceramics showed grains of irregular forms and different sizes varying from ~0.5 to 3 µm (Figure 2a). Variation in grain size may indicate fluctuation in chemical composition, varying from grain to grain. We deduced that such local disorder can diffuse phase transition [15,23,27]. The voids between the grains’ sharp edges resulted from the thermodynamic conditions. The occurrence of Ba, Ca, Ti, and O atoms was confirmed, and other elements were not detected using the EDS method (Figure 2b). 

The nominal chemical composition was: Ba—16.6%, Ca—3.4%, Ti—20%, and O—60%. Volatilization of the Ba, Ca, and Ti ions and their oxides was negligible at temperatures at which sintering was performed. Therefore, overall, the average concentration of the elements in BCT ceramics is to be close to the nominal. However, their local distribution could be non-homogeneous due to effective diffusion at high temperature. Hence, we conducted an EDS test for several areas and individual grains. The exemplar estimated compositions were: Ba—16.0%, Ca—3.2%, Ti—15.7%, O—65.1% for the polished cleaved surface and Ba—19.1%, Ca—2.9%, Ti—17.5%, O—60.5% for the un-polished cleaved surface of BCT ceramics. The experimental and nominal composition discrepancy exceeded accuracy frames. We note that Ti lines overlapped Ba lines in the spectrum, which might introduce additional errors in the estimation of concentration. The oxygen ion content was estimated with lower accuracy in accordance with the method and detector used, since it was obtained from summation up to 100%. The metal atom content ratio, Ba:Ca:Ti, was evaluated as an indicator of the chemical composition.

The Ba_0.83_Ca_0.17_TiO_3_ ceramics exhibited variation in composition, depending on the ceramics surface preparation, different from the nominal Ba:Ca:Ti = 41.5:8.5:50. Areas composed of a concentration of Ti ions higher than nominal, a concentration of Ba ions higher than nominal, and a similar content of Ba and Ti ions were found (Table 2). A unique precipitation of composition with ratio Ba:Ca:Ti = 54.9:17.8:27.3 also was found. Hence, we deduced the local fluctuation in chemical composition. Moreover, the tendency for an increased concentration of Ti ions in grain bulk and increased concentration of the Ba ions in grain surface or grain boundary cannot be excluded, since the polished ceramics exhibited the higher concentration of Ti ions.

We would like to notice that the applied final sintering temperature, 1673 K, was exactly the same as reported in the work by Lin et al. [16], 50 K higher than reported (1623 K) in the work by Zhu et al. [17], and 100 K higher than that reported by Tiwari at al. [24]. Hence, the diffusion in such high temperatures could be effective and responsible for homogenization of Ba and Ca ions, confirmed by the XRD test results, which indicated the random distribution of the Ca ions. We note that the XRD test exhibited the occurrence of a major tetragonal FE phase, which composition was close to the nominal Ba_0.83_Ca_0.17_TiO_3_. The content of the secondary phases was in the order of 2%. The penetration depth of the photon beam of the XRD test is much deeper than the electron beam penetration depth of the SEM test. Hence, the composition determined using XRD method can be considered as corresponding to the bulk of the ceramics grains. The SEM method corresponds, more or less, to the surface layer composition. Surface layers of ceramic grain termination with Ba–O might be deduced from the increased concentration of Ba detected for the un-polished surfaces of BCT ceramics (Table 2). The long-time heating of the perovskite materials, at elevated temperatures, can result in the diffusion of the ions from the bulk towards the surface, reconstruction of the surface, and occurrence of the Ruddlesden-Popper phases. On the other hand, we noticed that the Ruddlesden-Popper phases, which consist of multiple metal ion oxides in the surface layer, were not detected using the XRD test [32,33]. However, the chemical non-homogeneity on a nano-scale level cannot be excluded [34].

### 3.3. BDS

**Figure 3:**
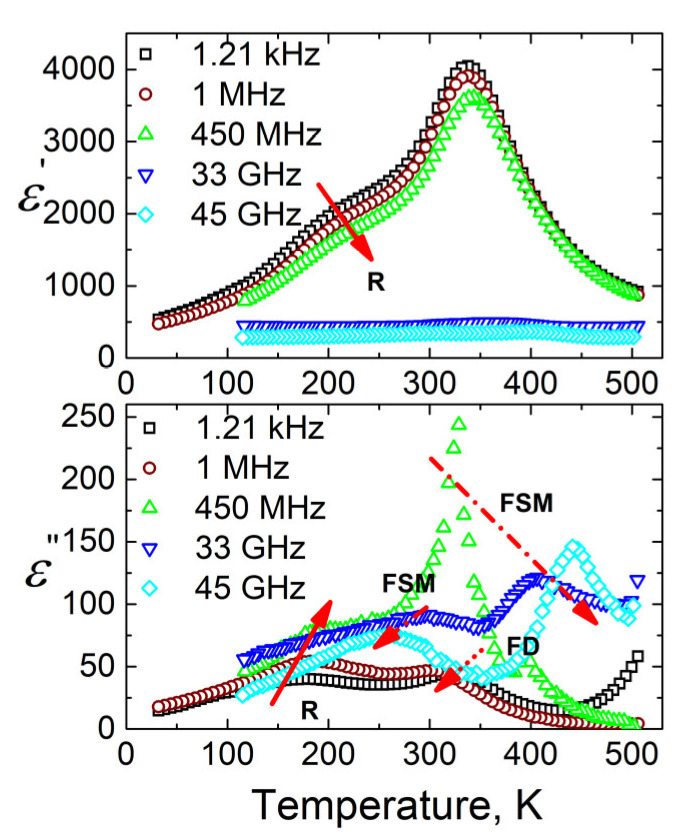
Temperature dependence of the real and the imaginary parts of complex dielectric permittivity ε* = ε’ − *i*ε” for Ba_0.83_Ca_0.17_TiO_3_ ceramics measured at several frequencies (R—relaxor, FSM—ferroelectric relaxational soft mode, FD—ferroelectric domains related dielectric dispersions).

The temperature dependencies of the real and the imaginary parts of dielectric permittivity for BCT ceramics are presented in Figure 3. Two dielectric anomalies can be clearly separated. The most pronounced dielectric anomaly is observed in vicinity of 340 K. At low frequencies (*f* < 1 MHz), the position of the real art of the dielectric permittivity maximum was frequency independent and only at higher frequencies (above 1 MHz); the maximum markedly shifted to the higher temperatures when the frequency increased. Such dielectric anomaly is typical for FE–PE phase transition [31]. Moreover, significant dielectric dispersion occurs for this material in microwaves. The step-like dielectric anomaly at lower temperatures (in 150–250 K range below 1 GHz) is strongly frequency dependent. Such an anomaly can be typical for relaxor or dipolar glass behaviour or FE domains [29]. 

These two anomalies visible in dielectric permittivity temperature dependence correlate to the sequence of phases reported for BCT with a Ca ion content of *x* = 0.15–0.20. The step-like anomaly can be related to the transition between the orthorhombic and the tetragonal FE phase observed in pure BaTiO_3_ [2]. The occurrence of this transition at ~100 K for *x* = 0.20, ~150 K for *x* = 0.165, and ~200 K for *x* = 0.15 was claimed by Zhu et al [17]. and Fu et al [22]. However, in the works [21,22,23,24,25], no X-ray investigations were performed below room temperature, and dielectric investigations were performed only in a narrow frequency range (below 1 MHz). The origin of the step-like anomaly will be discussed below by consideration of broadband dielectric spectra. The diffused peak, attributed to the FE–PE transition, occurred at a temperature about 60 K lower than the FE–PE transition, which occurred at ~400 K for the Curie-Weiss anomaly reported in the literature for the BCT [17,22]. The origins of such a marked shift, which indicates disturbed stability of the FE ordering, is not clear and needs further study. We would like to point out that, while XRD study proved the occurrence of one phase, SEM measurement exhibited fluctuation in local chemical composition. 

**Figure 4:**
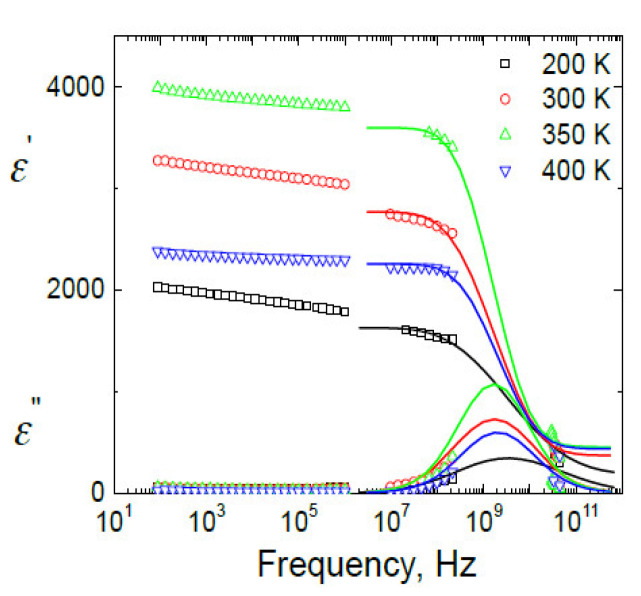
Frequency dependence of the real and the imaginary part of complex dielectric permittivity ε* = ε’ − *i*ε” for Ba_0.83_Ca_0.17_TiO_3_ ceramics measured at several temperatures. The lines denote the best fit according to Equation (1).

The frequency dependencies of complex dielectric permittivity for BCT ceramics are presented in Figure 4. The main dielectric dispersion occurs in frequency range 1 MHz–100 GHz. From these spectra the distributions of relaxation *f*(*τ*) were calculated according to the Tichonov regularisation method [35,36,37]:

(1) $$\varepsilon *\left(\nu \right)=\varepsilon_{\infty }+\Delta \varepsilon \int_{-\infty }^{\infty }\frac{f\left(\tau \right)dln\tau }{1+2\pi i\nu \tau }$$

**Figure 5:**
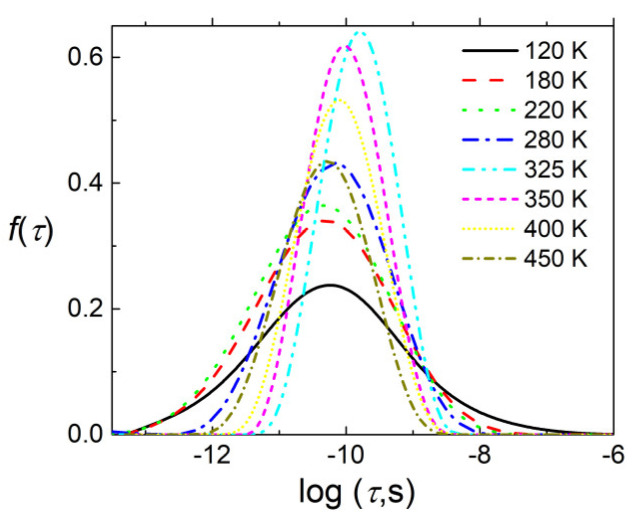
Distributions of relaxation times for BCT ceramics.

The distributions of relaxation times were calculated from the dielectric data in frequency range 1 MHz–40 GHz, while the dielectric dispersion below 1 MHz cannot be taken into account because it is very broad and its cutoff frequency is substantially below the low frequency limit (20 Hz). The obtained distributions of relaxation times are presented in Figure 5. The distribution of relaxation times shows the anomaly close to the FE phase transition temperature. This is typical behaviour for “order-disorder” FE phase transition [31]. 

**Figure 6:**
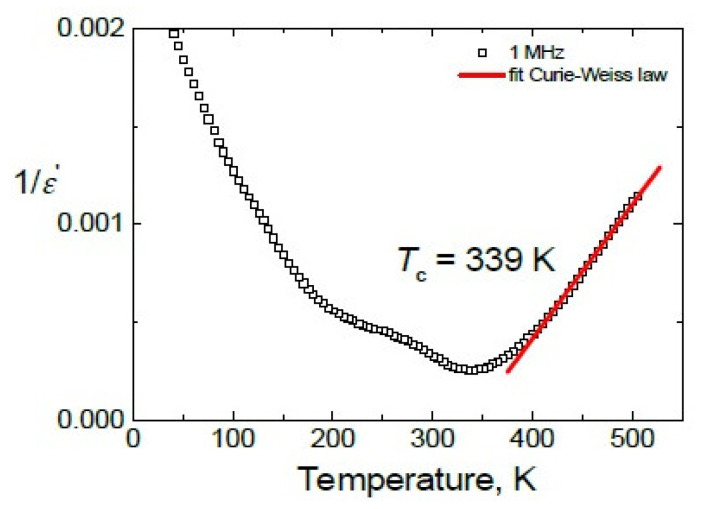
Temperature dependence of the reciprocal static dielectric permittivity.

The temperature dependence of the reciprocal static dielectric permittivity was plotted in Figure 6. The temperature dependence of the static dielectric permittivity was fitted with the Curie-Weiss law:

(2) $$\varepsilon^{'}=\varepsilon_{\infty }+\;\frac{C}{\left|\;T-T_{C\;}\right|}$$

only in the paraelectric phase because in the FE phase the temperature dependence of the dielectric permittivity was highly impacted by the second anomaly. The value *T*_C_ = 339 K was obtained.

**Figure 7:**
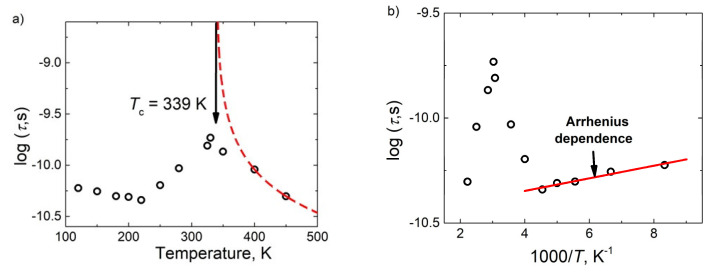
Temperature dependences of most probable relaxation time in linear temperature scale (**a**) and in reciprocal temperature scale (**b**).

Substantially below the FE phase transition temperature the distributions of relaxation times became very broad. From the distribution of relaxation times (Figure 5) the most probable relaxation time (the relaxation time at which the *f*(*τ*) magnitude is the biggest) was calculated (Figure 7).

The temperature dependence of the most probable relaxation time close to the FE–PE phase transition anomaly was also calculated according to the Curie-Weiss law [31]:

(3) $$\tau =\tau_{0}\;\frac{C}{\left|\;T-T_{C\;}\right|}.$$

Such behaviour of the relaxation time is typical for order-disorder FE phase transition [31]. The estimated *T*_c_ value was close to that obtained from the static dielectric permittivity fit (compare Figure 6 and Figure 7a).

At low temperatures, the most probable relaxation time follows the Arrhenius law:

(4) $$\tau \;=\;\tau_{0}\;\exp\;(E_{A}/\;k\;T\;)$$

Such behaviour of the most probable relaxation time is typical for FE domains or FE relaxors [29]. However, usually the dielectric dispersion typical for FE domains appears only at low frequencies (for example, below 1 MHz), while in this case of BCT the dielectric dispersion is observed also at higher frequencies, including microwaves (Figure 3 and Figure 4). Therefore, such features are typical for FE relaxors. The FE phase transition at 339 K is related with the cubic to tetragonal phase transition in pure BaTiO_3_ [2,17], while the relaxor behavior appears when the tetragonal to orthorhombic phase transition disappears in a BCT system [2,17]. Although the microscopic origin of these phase transitions is slightly different, they are both related to a shift of Ti^4+^ ions from their centrosymmetric positions [3]. Therefore, the crossover from FE to relaxor behavior is rather related to the diminishing of FE domain contributions and the occurrence of relaxation related to the reorientation of polar nanosized regions. The strong random electric fields are created due to substitution of Ba by Ca; obviously this is the main factor which determines the relaxor behaviour in the ceramics. 

## 4. Conclusions

Dielectric properties of Ba_1−*x*_Ca*_x_*TiO_3_ (*x* = 0.17) ceramics were studied in the wide frequency range 20 Hz–53 GHz. The order-disorder FE phase transition was revealed close to 339 K in the dielectric properties of ceramics. The behavior of the distributions of relaxation times, in vicinity of the FE phase transition temperature, is also typical for order-disorder FE phase transition. This confirms previous investigation results [3], that is, that the phase transitions in BaTiO_3_ related materials are rather mixed type, order-disorder and displacive. The order-disorder phase transition is mainly related to Ti^4+^ ions hopping. However, at lower temperatures (on cooling below 200 K), the most probable relaxation increased according to the Arrhenius law. At lower temperatures, the maximum of the imaginary part of dielectric permittivity versus temperature markedly shifted to higher temperatures with a frequency (from 125 K at 1.21 kHz to 300 K at 33 GHz). The behaviour was attributed to the FE relaxor. The relaxor behaviour is strongly related to the disappearance of the tetragonal to orthorhombic phase transition in the BCT system. The origin of the crossover from FE to relaxor behaviour in Ba_1−*x*_Ca*_x_*TiO_3_ (*x* = 0.17) ceramics is rather related to Ti^4+^ ions dynamics and strong random electric fields induced in the Ba sublattice due partial substitution of Ba by Ca. Thus, both FE and relaxor behaviors are strongly related to Ti^4+^ ions dynamics; non-ordered ions form polar nanoregions at lower temperatures.

## Figures and Tables

**Table 1:** The refined cell parameters determined for tetragonal *P*4*mm* phase Ba_1−*x*_Ca*_x_*TiO_3_ ceramics: *x*—Ca ion content, *a*, *b*, *c* and *α*, *β*, *γ* —crystal lattice parameters, and *V*—elementary cell volume.

Ca Content, *x*	0.17	0.18	0.20
*a* = *b* (Å)	3.98710(6)	3.98700(6)	3.98700(6)
*c* (Å)	4.00300(9)	4.00300(9)	4.00300(9)
*α =β = γ* (°)	90	90	90
*V* (Å^3^)	63.63555	63.63241	63.63237

**Table 2:** Exemplar atomic concentration (at. %) estimated for cleaved Ba_0.83_Ca_0.17_TiO_3_ ceramics surfaces. The nominal ratio is Ba:Ca:Ti = 41.5:8.5:50. The SEM signal was collected for large areas and for individual grains.

Content	Polished		Un-Polished		
	Area	Area	Area	Large Grain	Small Grain
	High Ti	Similar Ba and Ti	High Ba	High Ba	High Ba
Ba	39.1	45.9	49.1	46.5	47.1
Ca	6.3	9.1	7.8	9.3	9.3
Ti	54.6	45.0	43.1	43.9	43.6

## References

[1] Chan N.H., Sharma R.K., Smyth D.M. (1981). Nonstoichometry in undoped BaTiO_3_. J. Am. Ceram. Soc..

[2] Jona F., Shirane G. (1962). Ferroelectric Crystals.

[3] Hlinka J., Ospachuk T., Nuzhnyy D., Petzelt J., Kuzel P., Kadlec C., Vanek P., Ponomareva I., Bellaiche L. (2008). Coexistence of the phonon and relaxation soft modes in the terahertz dielectric response of tetragonal BaTiO_3_. Phys. Rev. Lett..

[4] Acosta M., Novak N., Rojas V., Patel S., Vaish R., Koruza J., Rossetti G.A., Rodel J. (2017). BaTiO_3_-based piezoelectrics: Fundamentals, current status and perspectives. Appl. Phys. Rev..

[5] Kozielski K., Wilk A., Bucko M.M., Banys J. (2020). A large piezoelectric strain recorded in BCT ceramics obtained by modified pechini method. Materials.

[6] He L.Q., Ji Y.C., Ren S., Zhao L., Luo H.Y., Liu C., Hao Y.S., Zhang L., Zhang L.X., Ren X.B. (2020). Large piezoelectric coefficient with enhanced thermal stability in Nb^5+^ doped Ba_0.85_Ca_0.15_Zr_0.1_Ti_0.9_O_3_ ceramics. Ceram. Int..

[7] Zheng T., Wu J.G., Xiao D.Q., Zhu J.G. (2018). Recent development in lead-free piezoelectric bulk materials. Prog. Mater. Sci..

[8] Shvartsman V.V., Dec J., Xu Z.K., Banys J., Keburis P., Kleemann W. (2008). Crossover from ferroelectric to relaxor behavior in BaTi_1−*x*_Sn*_x_*O_3_ solid solutions. Phase Transit..

[9] Nuzhnyy D., Petzelt J., Savinov M., Ostapchuk T., Bovtun V., Kempa M., Hlinka J., Buscaglia V., Buscaglia M.T., Nanni P. (2012). Broadband dielectric response of Ba(Zr,Ti)O_3_ ceramics: From incipient via relaxor and diffuse up to classical ferroelectric behavior. Phys. Rev. B.

[10] Canu G., Confalonieri G., Deluca M., Curecheriu L., Buscaglia M.T., Asandulesa M., Horchidan N., Dapiaggi M., Mitoseriu L., Buscaglia V. (2018). Structure-property correlations and origin of relaxor behaviour in BaCe*_x_*Ti_1−*x*_O_3_. Acta Mater..

[11] Filipic C., Kutnjak Z., Pirc R., Canu G. (2016). BaZr_0.5_Ti_0.5_O_3_: Lead free relaxor ferroelectric or dipolar glass. Phys. Rev. B.

[12] Lemanov V.V., Sotnikov A.V., Smirnova E.P., Weihnacht M., Kunze R. (1999). Perovskite CaTiO_3_ as an incipient ferroelectric. Solid State Commun..

[13] Pullar R.C., Zhang Y., Chen L., Yang S., Evans J.R.G., Salak A.N., Kiselev D.A., Kholkin A.L., Fierra V.M., Alford N.M. (2009). Dielectric measurements on a novel Ba_1−*x*_Ca*_x_*TiO_3_ (BCT) bulk ceramic combinatorial library. J. Electroceram..

[14] Liu F.W., Ren X.B. (2009). Large piezoelectric effect in Pb-free ceramics. Phys. Rev. Lett..

[15] Feliksik K., Kozielski L., Szafraniak-Wiza I., Goryczka T., Adamczyk-Habrajska M. (2019). Dielectric and impedance studies of (Ba,Ca)TiO_3_ ceramics obtained from mechanically synthesized powders. Materials.

[16] Lin T.-F., Lin J.-L., Hu C.-T., Lin I.-N. (1991). The microstructure developments and electrical properties of calcium-modified barium titanate ceramics. J. Mater. Sci..

[17] Zhu X.N., Zhang W., Chen X.M. (2013). Enhanced dielectric and ferroelectric characteristics in Ca-modified BaTiO_3_ ceramics. AIP Adv..

[18] Suresh P., Mathiyalagan P., Srikanth K.S. (2020). Structural, ferroelectric and photocatalysis performance of Ba_1−*x*_Ca*_x_*TiO_3_ ceramics. Ferroelectrics.

[19] Sharma P., Berwal N., Ahlawat N., Maan A.S., Punia R. (2019). Study of structural, dielectric, ferroelectric and magnetic properties of vanadium doped BCT ceramics. Ceram. Int..

[20] Krayzman V., Levin I., Woicik J.C., Bridges F., Nelson E.J., Sinclair D.C. (2013). Ca K-edge X-ray absorption fine structure in BaTiO_3_-CaTiO_3_ solid solutions. J. Appl. Phys..

[21] Panigrahi M.R., Panigrahi S. (2010). Diffuse phase transition and dielectric study in Ba_0.95_Ca_0.05_TiO_3_ ceramic. Phys. B Condens. Matter.

[22] Fu D., Itoh M., Koshihara S., Kosugi T., Tsuneyuki S. (2008). Anomalous Phase Diagram of Ferroelectric (Ba,Ca) TiO_3_ Single Crystals with Giant Electromechanical Respons. Phys. Rev. Lett..

[23] Shandilya M., Rai R., Zeb A., Kumar S. (2017). Modification of structural and electrical properties of Ca element on barium titanate nano-material synthesized by hydrothermal method. Ferroelectrics.

[24] Tiwari V.S., Pandey D., Groves P. (1989). The influence of a powder processing technique on chemical homogeneity and the diffuse phase transition behaviour of Ba_0.9_Ca_0.1_TiO_3_ ceramics. J. Phys. D. Appl. Phys..

[25] Wada S., Tabata K., Kakemoto H., Tsurumi T. (2004). Anomalous tructure and dielectric properties of BaTiO_3_-CaTiO_3_ system ceramic composites. J. Ceram. Soc. Jpn..

[26] Ellisade C., Ravez J. (2001). Ferroelectric ceramics: Defects and dielectric relaxations. J. Mater. Chem..

[27] Lee S., Randall C.A. (2008). A modified Vegard’s law for multisite occupancy of Ca in BaTiO_3_-CaTiO_3_ solid solutions. Appl. Phys. Lett..

[28] Levin I., Krayzman V., Woicik J.C. (2013). Local-structure origins of the sustained Curie temperature in (Ba,Ca)TiO_3_ ferroelectrics. Appl. Phys. Lett..

[29] Macutkevic J., Banys J., Grigalaitis R., Vysochanskii Y. (2008). Asymmetric phase diagram of CuInP_2_(S*_x_*Se_1−*x*_)_6_ crystals. Phys. Rev. B.

[30] Degen T., Sadki M., Bron E., König U., Nénert G. (2014). The HighScore suite. Powder Diffr..

[31] Grigas J. (1996). Microwave Dielectric Spectroscopy of Ferroelectrics and Related Materials.

[32] Szot K., Pawelczyk M., Herion J., Freiburg C., Albers J., Waser R., Hulliger J., Kwapulinski J., Dec J. (1996). Nature of the surface layer in ABO_3_-type perovskites at elevated temperatures. Appl. Phys. A.

[33] Mukhopadhyay S.M., Chen T.C.S. (1995). Surface chemical states of barium titanate: Influence of sample processing. J. Mater. Res..

[34] Molak A., Paluch M., Pawlus S., Klimontko J., Ujma Z., Gruszka I. (2005). Electric modulus approach to analysis of the electric relaxation in highly conducting (Na_0.75_Bi_0.25_)(Mn_0.25_Nb_0.75_)O_3_ ceramics. J. Phys. D Appl. Phys..

[35] Schafer E., Sternin R., Stannarius M., Arndt M., Kremer F. (1996). Novel approach to the analysis of broadband dielectric spectra. Phys. Rev. Lett..

[36] Tikhonov A.N. (1965). Ill-posed problem of linear algebra and stable methods for solving them. Dokl. Akad. Nauk SSSR.

[37] Macutkevic J., Banys J., Matulis A. (2004). Determination of distribution of the relaxation times from dielectric spectra. Nonlinear Anal. Model. Control.

